# Comparison of polymerization shrinkage of a new bulk‐fill flowable composite with other composites: An in vitro study

**DOI:** 10.1002/cre2.656

**Published:** 2022-09-05

**Authors:** Somayeh Khoramian Tusi, Hajar Hamdollahpoor, Maryam Mohammadi Savadroodbari, Mahmood Sheikh Fathollahi

**Affiliations:** ^1^ Department of Pediatric Dentistry Alborz university of Medical Sciences Karaj Iran; ^2^ Student Research Committee Alborz University of Medical Sciences Karaj Iran; ^3^ Department of Operative Dentistry Alborz University of Medical Sciences Karaj Iran; ^4^ Rajaie Cardiovascular Medical and Research Center Iran University of Medical Sciences Tehran Iran

**Keywords:** bulk‐fill, composite resins, flowable hybrid composite, polymerization, shrinkage

## Abstract

**Objective:**

Since composites still face a critical problem called polymerization shrinkage and bulk‐fill composites have reported acceptable results for this issue, this study aims to assess the polymerization shrinkage of a new bulk‐fill flowable composite (G‐aenial bulk injectable [GBI]) and compare it to other bulk‐fill and conventional composites.

**Materials and Methods:**

In this in vitro study, 25 composite discs were fabricated using three bulk‐fill and two conventional composites. They were bonded to a microscopic slide and were covered by a coverslip. This assembly was transferred to a linear variable differential transformer and composite samples were cured from underneath the slides. Dimensional changes formed in composite samples were recorded. Data were analyzed using analysis of variance followed by post hoc Tukey's and Dunnett's tests.

**Results:**

The groups were significantly different regarding polymerization shrinkage. G‐aenial bulk injectable and G‐aenial universal flo showed significantly higher polymerization shrinkage than other composites at 30, 60, and 1800 s after light irradiation, while X‐tra fil and Filtek Z250 showed the lowest polymerization shrinkage at the aforementioned time points.

**Conclusion:**

According to the results, the new composite had polymerization shrinkage similar to the conventional one. Bulk‐fill composites reported similar or lower shrinkage to conventional composites.

## Clinical significance

Polymerization shrinkage can lead to treatment failure by causing secondary caries, enamel cracks, posttreatment pain, and pulpitis. Since the type of material has a role in the amount of this shrinkage, using composites with lower polymerization shrinkage causes restorations with increased longevity.

## 1 INTRODUCTION

Since late 1990 using composites has significantly grown up due to their biocompatibility (Habib et al., 2016) and constant improvements in their esthetic and mechanical features. Today, composites are the first choice for lots of direct restorations in dentistry and clinical studies have reported their positive results with increased longevity (Park et al., 2021). These materials cause minimum loss of tooth tissue and give maximum strength while meeting esthetic and functional needs in restorative dentistry (Ersen et al., 2020). Another advantage is that, unlike amalgam, composites allow us to manipulate them for a long time or to restore the former restoration and they also have a long working time (Haugen et al., 2020).

Resin composites are a composition of the organic matrix, inorganic fillers, and silane coupling agent (Moldovan et al., 2019). The organic matrix includes diverse monomers, such as bisphenol A‐glycidyl methacrylate (BisGMA), urethane dimethacrylate, triethylene glycol dimethacrylate (TEGDMA), dimethylaminoethyl methacrylate, and also different additives, including photopolymerization initiators, inhibitors, accelerators, and stabilizers (Baroudi et al., 2007; Braga et al., 2005; Ferracane, 2008). Inorganic fillers determine most of the mechanical properties, visual features, and radioopacity of composites. The silane coupling agent links the two main parts (matrix and fillers) of resin composites (Habib et al., 2016; Moldovan et al., 2019).

Dental composites formula has been improving since their introduction to dentistry and has led to the production of satisfying materials. Despite these improvements in all physical and mechanical features during recent years, they still face a critical limitation called polymerization shrinkage (Meereis et al., 2018). The best function of resin materials is dependent on the correct polymerization of their components, which is explained by the monomer's conversion into polymers. This conversion is accompanied by a reduction of material volume because a polymer occupies less volume than monomers. The effect of this conversion has been known as polymerization shrinkage (Soares et al., 2017). This shrinkage is mostly affected by the formulation and properties of restorative materials, adhesion, flow on the free surface, and polymerization kinetics (Sampaio et al., 2017). Dental composites usually undergo 1%–6% volumetric shrinkage according to their formula and curing conditions (Rizzante et al., 2019; Soares et al., 2017). This shrinkage generates stress of about 5–15 MPa (Kaisarly & El Gezawi, 2016) at the interface of the tooth and restorative material causes gaps and consequently microleakage (Atai et al., 2005; Gerula‐Szymańska et al., 2020). Possible results are posttreatment pain and sensitivity, (Kleverlaan & Feilzer, 2005) restoration with discolored margins, cuspal deflection, recurrent caries, and pulpal effects (Kaisarly et al., 2021; Zorzin et al., 2015). This stress can overcome the tensile strength of enamel and cause enamel cracks (Kaisarly & El Gezawi, 2016; Kaisarly et al., 2021). Although measuring the correlation between polymerization stress and treatment failures is difficult, in vitro studies have shown the necessity of polymerization stress management for treatment success (Meereis et al., 2018). Practical strategies like curing protocols, using low viscosity liners and layering techniques have been suggested for reducing polymerization stress (Zorzin et al., 2015). The layering technique allows light to penetrate perfectly through composite layers, complete curing takes place and polymerization shrinkage decreases (Al Aqil et al., 2020). Despite the effects of this technique on the reduction of polymerization stress, (Sampaio et al., 2017) this method faces some drawbacks like elongation of treatment, void formation, and the possibility of contamination or debonding of layers (A. Correia et al., 2020; Park et al., 2021).

In recent years, a new group of composites named bulk‐fill composites has been introduced for facilitating restorative treatments. These materials cause restorative treatments to be shorter and less technique‐sensitive (Sampaio et al., 2017). Producers claim that these composites can be cured and polymerized to the depth of 4–5 mm and at the same time they can maintain polymerization stress at a low level (Tauböck et al., 2017; Zorzin et al., 2015). According to manufacturers, increased curable depth of composite does not diminish its quality. Meanwhile, the time required to place the filling is reduced by up to 30% (Gerula‐Szymańska et al., 2020).

Bulk‐fill composites manufacturers gain deeper polymerization and reduced stress through different strategies. Some of them use additional or more efficient photoinitiators and others try to enhance light transmission through the composites by some approaches, such as reducing filler content (Soares et al., 2017).

Based on the fact that bulk‐fill composites are considered fairly new in dentistry, further studies are needed to analyze these composites, but according to published articles, they can be used satisfactorily as stress reducer materials (Meereis et al., 2018). It has been reported that bulk‐fill composites have less or the same polymerization shrinkage, polymerization stress, cuspal deflection, and marginal gaps in comparison to conventional ones (da Silva Pereira et al., 2020). Also, their adequate clinical behavior after 3 and 5 years has been stated (Marovic et al., 2015; Soares et al., 2017).

Like conventional composites, these composites are available in high and low viscosities (Santini et al., 2012). Different viscosities are because of filler content, which has a direct effect on the modulus of elasticity (Jang et al., 2015; Kaisarly et al., 2021). Flowable composites have less filler content and packable composites have more filler content (Kim et al., 2015). In composites with low viscosity, stress reduction is gained by the low modulus of elasticity and it leads to polymerization shrinkage to be compensated through the deformation of restorative material (Kaisarly et al., 2021; Labella et al., 1999; Tauböck et al., 2019; Van Ende et al., 2017).

Using flowable bulk‐fill composites has the advantage of better material adjustment to the cavity walls and polymerization stress reduction due to low modulus of elasticity (T. C. Correia et al., 2017; Soares et al., 2017). It was reported that flowable bulk‐fill composites had better marginal seals compared to packable bulk‐fill composites (Orłowski et al., 2015). These materials are so practical in cavities with a small size or complicated design (Gerula‐Szymańska et al., 2020; Park et al., 2021).

Since bulk‐fill composites are recently introduced and therefore there are insufficient studies and also contradictory results, it is critical to do more research about these materials (Abbasi et al., 2018; Al Aqil et al., 2020; Cidreira Boaro et al., 2019).

A new flowable bulk‐fill composite named “G‐aenial bulk injectable” (GBI) from GC Company is claimed to have satisfying features, such as low polymerization shrinkage. Since this composite has not been studied yet, the present study aimed to assess its polymerization shrinkage and compare it to other bulk‐fill and conventional composites.

The null hypotheses tested was: There would be no difference in polymerization shrinkage of the new bulk fill flowable composite (GBI) and other composites.

## 2 MATERIALS AND METHODS

In this in vitro study, five types of commercial composites, including two conventional ones: GUF and Filtek Z250 (Z250), and three bulk‐fill ones: G‐aenial bulk injectable (GBI), X‐tra base (XB), and X‐tra fil (XF) were assessed.

Characteristics of composites used in the current study are demonstrated in Table 1.

**Table 1:** Characteristics of resin composites used in this study

Code	Commercial brand	Type of composite	Manufacturer	Composition	Filler percentage	Color	Batch number
1	G‐aenial bulk injectable	Bulk‐fill flowable composite	GC Corporation, Tokyo, Japan	N/A	N/A	A2	1901281
2	X‐tra base	Bulk‐fill flowable composite	Voco (Cuxhaven, Germany)	UDMA Bis‐EMA	75% wt 60 vol	U	2002369
3	X‐tra fil	Bulk‐fill paste composite	Voco (Cuxhaven, Germany)	Bis‐GMA UDMA TEGDMA	86% wt	U	1175
4	G‐aenial universal flo	Nano‐hybrid flowable	GC Corporation, Tokyo, Japan	UDMA Bis‐MEPP TEGDMA	69% wt 50 vol	A2	190603A
5	Filtek Z250	Microhybrid restorative	3M ESPE (St. Paul, MN)	Bis‐GMA UDMA Bis‐EMA	82% wt 60 vol	A2	NA95737

The sample size was calculated to be five samples in each of the five groups using one‐way analysis of variance (ANOVA) and the power analysis feature of PASS II software (NCSS, LLC, Kaysville, UT, USA), assuming *α* = .05, *β* = .2, standard deviation (SD) = 0.04, and effect size = 0.28 according to a study by Abbasi et al. (2018).

**Figure 1:**
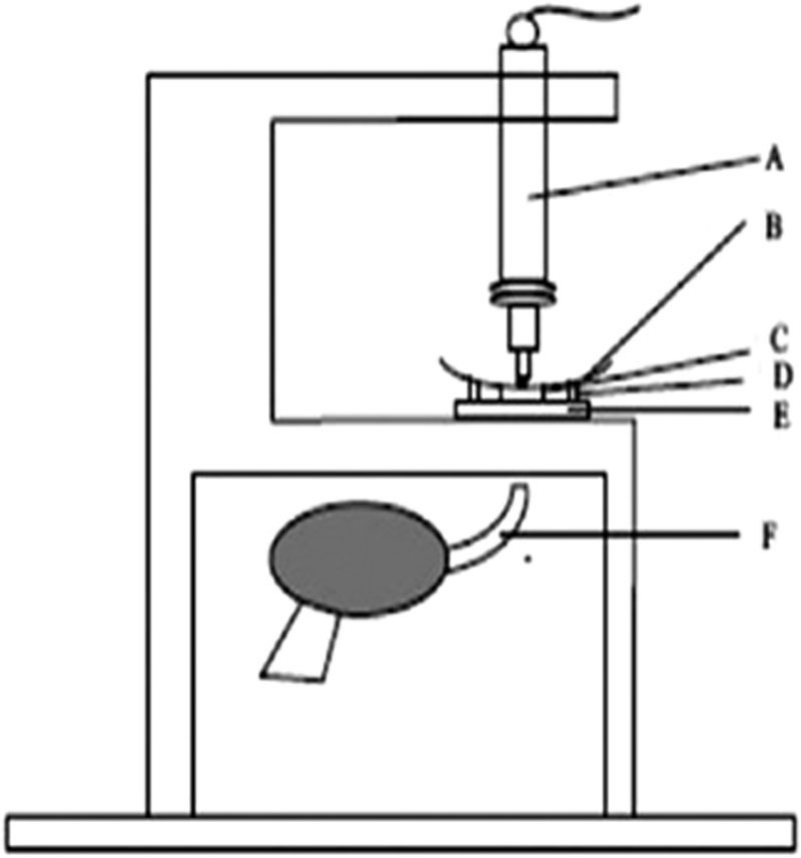
Schematic picture of bonded disc method instrument; (A) LVDT transducer, (B) cover slip, (C) disk specimen, (D) brass ring, (E) rigid glass plate, (F) light source. LVDT, linear variable differential transformer.

In the present study, the bonded‐disc method, which was primarily presented by Watts and Cash (Kaisarly & El Gezawi, 2016) was used for evaluating polymerization shrinkage. Figure 1 shows the parts used in this method schematically. In this method, dimensional changes of samples caused by polymerization shrinkage are analyzed by a linear variable differential transformer (LVDT) device.

**Figure 2:**
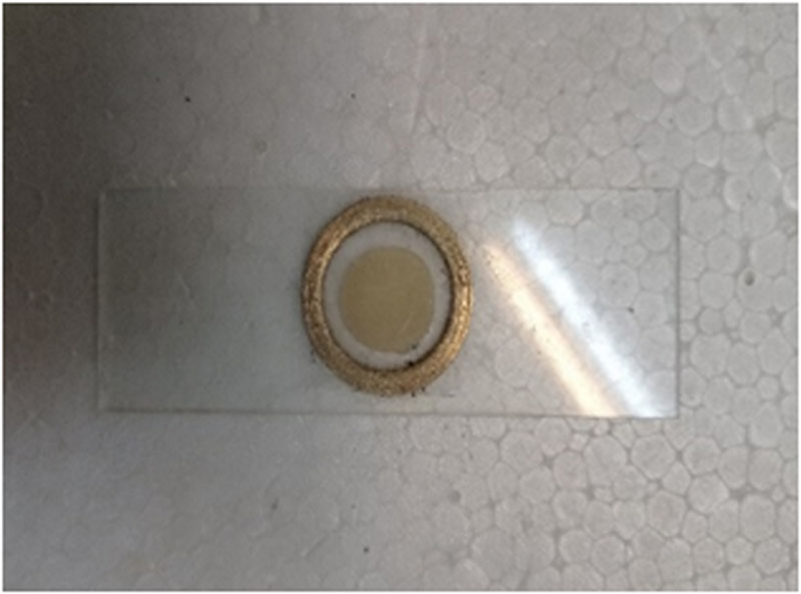
A sample of a packed composite disc.

In this study, five samples were prepared using each kind of composite (*N* = 25); each sample contained 0.2 gr composite, which was formed in the shape of a disc and then was applied in a microscopic slide measuring 1 × 25 × 75 mm^2^, at the center of a brass ring with the diameter of 16 mm and height of 1 mm in a way that there was a free space around the disc (Figure 2).

**Figure 3:**
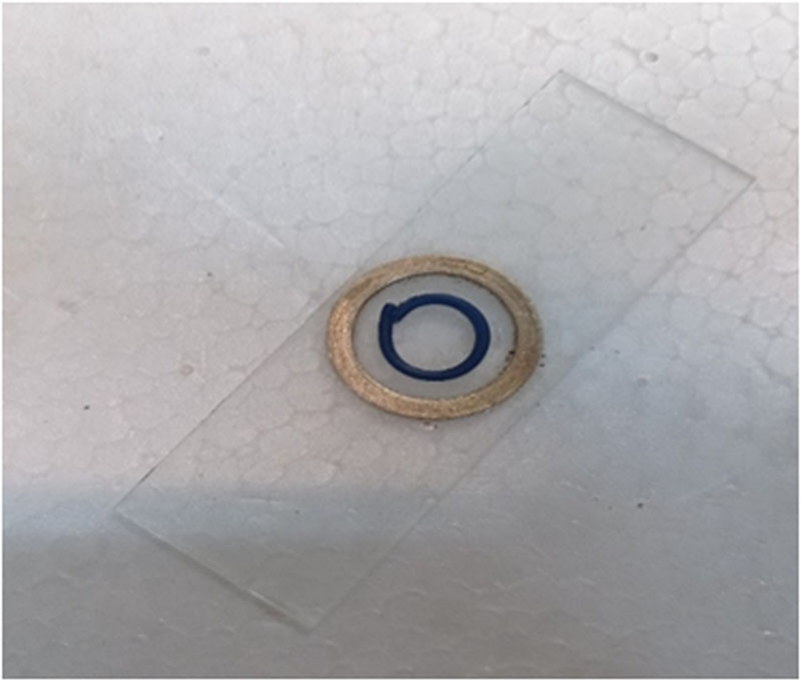
Paraffin ring for restricting flowable composites.

The upper surface of the microscopic slide was grit‐blasted with 50 µm alumina powder for better bonding of composite samples. For low viscosity composites, a paraffin ring was used to restrict the samples to a certain size (Figure 3).

In the next step, the composite was packed into the brass ring using another slide. A cover slip with a dimension of 22 × 22 and 0.13 mm thickness (borosilicate glass, thickness No. 0; VWR International Ltd, Randor, PA, USA) was used to cover the composite sample and brass ring, then the assembly was placed on the jig of the LVDT (RDP Electronics Ltd., Wolverhampton, UK).

Jig is composed of a horizontal aluminum plate for placing the sample and two stainless clamps for maintaining the microscopic slide. The transducer was positioned in touch with the center of the coverslip and samples were cured using a light‐curing unit (OptiPlex 500–Kerr, Orange, CA, USA) for 40 s from underneath the microscopic slide. Light irradiation of the light‐curing unit was verified by a radiometer (L.E.D Kerr, USA) constantly.

Upon light irradiation, the composite discs underwent shrinkage and the following flexure, which were monitored by the transducer with an accuracy of 0.01 µm. The changes were recorded by a recorder on a computer for 30 min. Since the shrinkage is mainly vertical in this method, only the changes in the thickness of the samples were recorded and relative linear shrinkage was considered approximately equivalent to relative volumetric shrinkage:

(1) $$\frac{∆L}{L_{0}}≅\frac{∆V}{V0}.$$

The shrinkage strain rate was calculated by numerical differentiation of the shrinkage strain data with respect to time (Atai et al., 2005).

Data were expressed as mean ± SD. One‐way ANOVA followed by post‐hoc Tukey's and Dunnett's tests were used to compare the polymerization shrinkage of composites at 1, 30, 60, and 1800 s following the onset of irradiation. Kolmogorov–Smirnov test was used to assess the normality assumption. The test indicated that the normality presumption was not violated (*p* > .05). Levene's test of equality of error variances across the five groups was also approved (*p* > .05). For the statistical analysis, the statistical software SPSS version 22.0 for windows (IBM SPSS Inc., Chicago, IL, USA) was used. All *p* values were two‐tailed, with statistical significance defined by *p* ≤ .05.

## 3 RESULTS

Table 2 shows “minimum, maximum, mean, and SD” for polymerization shrinkage of resin composites at 1, 30, 60, and 1800 s following irradiation onset. Since one‐way ANOVA demonstrated a significant difference in the polymerization shrinkage of different composites at 1, 30, 60, and 1800 s (*p* < .05), post‐hoc Tukey's test and post hoc Dunnett's test were used to compare the polymerization shrinkage of composites.

**Table 2:** Descriptive characteristics of polymerization shrinkage (µm) of composite resins at 1, 30, 60, and 1800 s following the onset of irradiation

Group (*n* = 5)	Composite	Time	Minimum	Maximum	Mean	Standard deviation
1	G‐aenial bulk injectable	1 s	0.125	0.432	0.291	0.140
		30 s	3.135	3.544	3.401	0.155
		60 s	3.578	3.999	3.837	0.155
		1800 s	4.078	4.578	4.360	0.180
		Rate	0.102	0.114	0.107	0.005
2	X‐tra base	1 s	0.125	0.545	0.348	0.188
		30 s	2.556	2.965	2.799	0.150
		60 s	2.783	3.192	3.026	0.153
		1800 s	3.113	3.453	3.315	0.134
		Rate	0.080	0.093	0.085	0.005
3	X‐tra fil	1 s	0.227	0.954	0.529	0.305
		30 s	0.943	2.068	1.570	0.489
		60 s	1.034	2.238	1.711	0.529
		1800 s	1.409	2.624	2.024	0.510
		Rate	0.019	0.056	0.036	0.015
4	G‐aenial universal flo	1 s	0.557	0.909	0.727	0.130
		30 s	3.590	3.896	3.712	0.141
		60 s	3.874	4.271	4.024	0.180
		1800 s	4.271	4.828	4.487	0.227
		Rate	0.099	0.111	0.103	0.005
5	Filtek Z250	1 s	0.250	0.738	0.411	0.200
		30 s	1.852	2.011	1.915	0.061
		60 s	2.045	2.215	2.106	0.068
		1800 s	2.181	2.601	2.404	0.150
		Rate	0.041	0.058	0.052	0.007

Results of pairwise comparison at the first second following onset of irradiation are shown in Table 3. GUF showed the highest (0.727% ± 0.130%) and GBI showed the lowest polymerization shrinkage (0.291% ± 0.140%) at this time. As presented, the polymerization shrinkage of GUF was significantly higher than GBI and XB (*p* ≤ .053). Shrinkage difference was not noticeable among other groups.

**Table 3:** Pairwise comparisons of the polymerization shrinkage of composites at the first second following the onset of irradiation

First second	GBI	XB	XF	GUF	Z250
GBI	*				
XB	*p* = .991	*			
XF	*p* = .367	*p* = .622	*		
GUF	*p* = .389	*p* = .053	*p* = .547	*	
Z250	*p* = .877	*p* = .987	*p* = .885	*p* = .138	*

Abbreviations: GBI, G‐aenial bulk injectable; GUF, G‐aenial universal flo; XB, X‐tra base; XF, X‐tra fil; Z250, Filtek Z250.

Table 4 presents the results of a pairwise comparison of the composite resins at 30 s after the onset of irradiation. The lowest polymerization shrinkage was for XF (1.570% ± 0.489%), which had a significant difference from other composites (*p* < .001) but Z250. GUF and GBI showed significant higher polymerization shrinkage in comparison to other composites (*p* ≤ .008).

**Table 4:** Pairwise comparisons of the polymerization shrinkage of composites at 30 s following the onset of irradiation

30 s	GBI	XB	XF	GUF	Z250
GBI	*				
XB	*p* = .008	*			
XF	*p* < .001	*p* < .001	*		
GUF	*p* = .311	*p* < .001	*p* < .001	*	
Z250	*p* < .001	*p* < .001	*p* = .222	*p* < .001	*

Abbreviations: GBI, G‐aenial bulk injectable; GUF, G‐aenial universal flo; XB, X‐tra base; XF, X‐tra fil; Z250, Filtek Z250.

Tables 5 and 6 demonstrate the results of a pairwise comparison of composite resins at 60 and 1800 s following light‐curing onset. At both time points, GUF experienced the highest polymerization shrinkage (4.024% ± 0.180% and 4.487% ± 0.227%, respectively) with an insignificant difference with GBI. XF showed the lowest polymerization shrinkage (1.711% ± 0.529% and 2.024% ± 0.510%, respectively) with an insignificant difference with Z250. XB had a significant lower polymerization shrinkage in comparison to GUF and GBI, and a significant higher polymerization shrinkage compared to XF and Z250.

**Table 5:** Pairwise comparisons of the polymerization shrinkage of composites at 60 s following the onset of irradiation

60 s	GBI	XB	XF	GUF	Z250
GBI	*				
XB	*p* = .001	*			
XF	*p* < .001	*p* < .001	*		
GUF	*p* = .809	*p* < .001	*p* < .001	*	
Z250	*p* < .001	*p* < .001	*p* = .181	*p* < .001	*

Abbreviations: GBI, G‐aenial bulk injectable; GUF, G‐aenial universal flo; XB: X‐tra base; XF, X‐tra fil; Z250, Filtek Z250.

**Table 6:** Pairwise comparisons of the polymerization shrinkage of composites at 1800 s following the onset of irradiation

1800 s	GBI	XB	XF	GUF	Z250
GBI	*				
XB	*p* < .001	*			
XF	*p* < .001	*p* < .001	*		
GUF	*p* = .948	*p* < .001	*p* < .001	*	
Z250	*p* < .001	*p* < .001	*p* = .233	*p* < .001	*

Abbreviations: GBI, G‐aenial bulk injectable; GUF, G‐aenial universal flo; XB, X‐tra base; XF, X‐tra fil; Z250, Filtek Z250.

Table 7 shows the shrinkage strain rate comparison among resin composites. As presented, XF had a significantly lower shrinkage speed compared to other composites (0.036 ± 0.015). GUF and GBI showed higher shrinkage strain rates compared to other composites (*p* ≤ .017).

**Table 7:** Comparison of the shrinkage strain rate of composite resins

RATE	GBI	XB	XF	GUF	Z250
GBI	*				
XB	*p* = .003	*			
XF	*p* < .001	*p* < .001	*		
GUF	*p* = .922	*p* = .017	*p* < .001	*	
Z250	*p* < .001	*p* < .001	*p* = .046	*p* < .001	*

Abbreviations: GBI, G‐aenial bulk injectable; GUF, G‐aenial universal flo; XB, X‐tra base; XF, X‐tra fil; Z250, Filtek Z250.

Table 8 presents the comparison of polymerization shrinkage of the new studied composite “GBI” and other composites. At the first second after the onset of irradiation, this composite had a significant lower polymerization shrinkage compared to GUF (*p* = .010) but had no significant difference from others. At 30, 60, and 1800 s after light‐curing, it had significant higher polymerization shrinkage compared to XF, Z250, and XB (*p* ≤ .004). The difference between this composite and GUF was not significant. The shrinkage speed of this composite was significantly higher than XF, Z250, and XB (*p* < .001) but similar to GUF.

**Table 8:** Comparison of the polymerization shrinkage and shrinkage strain rate of GBI and other composites

	1 s	30 s	60 s	1800 s	Rate
XB	*p* = .976	*p* = .004	*p* < .001	*p* < .001	*p* = .001
XF	*p* = .222	*p* < .001	*p* < .001	*p* < .001	*p* < .001
GUF	*p* = .010	*p* = .182	*p* = .654	*p* = .878	*p* = .829
Z250	*p* = .754	*p* < .001	*p* < .001	*p* < .001	*p* < .001

Abbreviations: GBI, G‐aenial bulk injectable; GUF, G‐aenial universal flo; XB, X‐tra base; XF, X‐tra fil; Z250, Filtek Z250.

**Figure 4:**
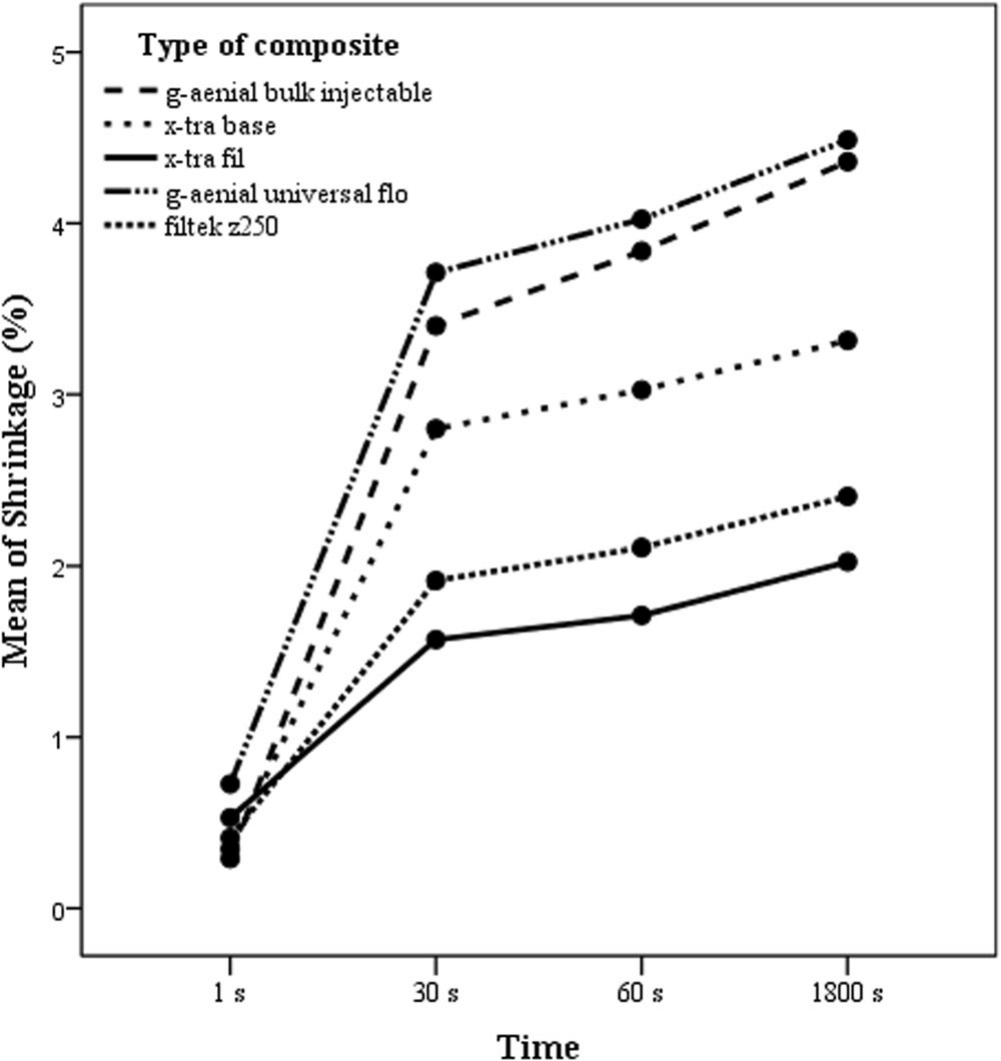
Schematic comparison of polymerization shrinkage of resin composites at 1, 30, 60, and 1800 s following irradiation onset.

Figure 4 presents the comparison of polymerization shrinkage among studied resin composites schematically.

## 4 DISCUSSION

In the present study in which the polymerization shrinkage of resin composites was reported until 30 min after the onset of irradiation, the groups were significantly different. The assessed shrinkage ranged from 0.4 to 2.4 for packable composites and 0.2–4.4 for flowable ones which are in accordance with the results of Rizzante et al. (2019) that reported 1%–3% shrinkage for packable composites and up to 6% shrinkage for flowable ones.

For measuring polymerization shrinkage, we used the bonded‐disc method, which was first developed in 1991 by Watts and Cash and is the most common way to measure composite shrinkage (Meereis et al., 2018). For evaluating polymerization shrinkage in this method, changes in the vertical dimension of composites (linear shrinkage) are quantified; so since linear shrinkage is calculated and then it converts into volumetric shrinkage, the shrinkage can be reported less than its actual amount and this can be a drawback for this method. The advantages of this technique are adjustment of a defined c‐factor, complete curing of composite samples due to their low thickness, allowing the use of different light intensities at different temperatures and ease and convenience of use (Abbasi et al., 2018; Kaisarly & El Gezawi, 2016).

The polymerization shrinkage of resin composites depends on the amount, type, and size of fillers. Generally, an increase in filler content decreases available monomers for curing reaction and consequently leads to lower polymerization shrinkage. However high filler content causes a higher modulus of elasticity and it rises polymerization stress according to Hooke's law (Labella et al., 1999; Tauböck et al., 2019). So it is important to know that composites with higher polymerization shrinkage do not necessarily cause higher polymerization stress because the whole generated shrinkage does not convert into stress; polymer can rearrange and release some stress. This stress is affected by the shape and size of the tooth cavity as well as the amount of polymerization shrinkage and modulus of elasticity (Van Ende et al., 2017).

Other factors affecting polymerization shrinkage include the type of resin matrix, concentration of monomer, and type of photoinitiators (Ferracane, 2008). For example, diluent concentration in resin matrix has an effect on the shrinkage of composite resins; recent studies have reported that an increased proportion of TEGDMA compared to BisGMA in experimental composite resins caused more contraction stress related to more polymerization shrinkage due to increased conversion. Since diluent monomers (like TEGDMA) mostly have less molecular weight than host monomers, the density of polymerizable carbon double bonds is increased and it can lead to more polymerization shrinkage (Baroudi et al., 2007; Braga et al., 2005).

According to the results of this study, the new composite, GBI, had significant higher polymerization shrinkage compared to XF, Z250, and XB but it had similar shrinkage to the conventional flowable composite (GUF) at 30, 60, and 1800 s, so the null hypothesis was partially rejected. This result can illustrate that the amount of polymerization shrinkage in bulk‐fill composites is highly dependent on the commercial brand of the used composite and its composition. Cidreira Boaro et al. (2019) reported different functions for various bulk‐fill composites due to their brands.

However, GBI had similar shrinkage compared to GUF, the company suggests some advantageous features for GBI. According to the company's claim, it can be used up to 4 mm depth of cure without the need for capping or veneering with other composites. Besides easy one‐step filling, full coverage silane coating of nanoparticles facilitates excellent adaptation to the cavity and provides extraordinary wear‐resistance and gloss retention. Further studies are required to investigate other features of this composite, such as marginal seal and polymerization stress.

XF had the lowest and GUF had the highest polymerization shrinkage at 30, 60, and 1800 s after the onset of light‐curing. In both composite groups with high and low viscosity, bulk‐fill composites had similar or less polymerization shrinkage than conventional ones, which is in agreement with the results of the other study (Abbasi et al., 2018).

In this study as in some other studies (Rizzante et al., 2019; Tauböck et al., 2019) a strong correlation was observed between filler content and polymerization shrinkage. The composite with the lowest polymerization shrinkage had the highest filler content (86% wt/70% vol) and the composite with the highest polymerization shrinkage had the lowest filler content (69% wt/50% vol).

Based on the results of this study, composites with high viscosity had lower polymerization shrinkage, which confirms the results of other studies (Jang et al., 2015). This can explain by the amount of filler content. Since low‐viscosity composites have lower inorganic fillers and higher resin matrix, and shrinkage is a result of monomers conversion into polymers, they will experience higher shrinkage (Jang et al., 2015).

Low‐viscosity bulk‐fill composites showed various results. XB had significantly lower polymerization shrinkage compared to conventional flowable composite but lower polymerization compared to conventional packable composite. Likewise, Jang et al. (2015) showed that bulk‐fill flowable composites had lower polymerization shrinkage than conventional flowable composites and higher polymerization shrinkage than conventional packable ones. However, the other bulk‐fill flowable composite (GBI) had similar shrinkage to conventional flowable composite, which can be related to these composites' content of resin matrix. XB had the lowest shrinkage among flowable composites (3.3%), similarly, T. C. Correia et al. (2017) reported in their study that XB caused the lowest marginal gap.

For reaching a 4 mm depth of cure in bulk‐fill composites, producers of these types of composites with low viscosity have taken advantage of the fact that decreasing filler content or increasing its size causes enhanced translucency (Marovic et al., 2015). When using these composites, care must be taken that it is required to use a covering layer of a packable conventional composite over the low‐viscosity composite to enhance its mechanical features. This step not only reinforces surface hardness but also is needed to stop water absorption because bulk‐fill composites are more prone to water deterioration compared to conventional ones (Jang et al., 2015).

XF had the lowest polymerization shrinkage. Abbasi et al. (2018) who assisted polymerization shrinkage of five bulk‐fill composites also reported XF as the composite with the lowest polymerization shrinkage. According to the manufacturer, this composite contains a mixture of multihybrid fillers and a new initiator for lowering polymerization shrinkage.

The highest recorded polymerization shrinkage was for GUF. This composite is a highly filled composite with low viscosity, which contains TEGDMA. This monomer has high reactivity, which increases double bond monomers conversion and consequently increases polymerization shrinkage (Abbasi et al., 2018; Marovic et al., 2015).

XF had a considerably lower polymerization rate than other composites. This composite includes a high amount of BisGMA. This monomer is susceptible to making secondary hydrogen bonds with adjacent molecules due to containing central phenol circles and hydroxyl bonds. The result is a high viscosity and lower diffusion of monomers during polymerization, which explains the low rate of polymerization in this composite (Santini et al., 2012).

According to the results of other studies, polymerization shrinkage is directly related to generated polymerization stress (Atai et al., 2005). Faster polymerization shrinkage indicates that resin composite reaches its gel point quicker and it accelerates its setting rather than giving it time to flow. A higher polymerization rate leads to a faster increase in modulus of elasticity, which generates more stress (Kleverlaan & Feilzer, 2005). So we can come to the conclusion that XF might cause lower polymerization stress compared to other composites in the same circumstances.

The rapid rise of polymerization shrinkage of studied resin composites is shown at the beginning seconds, which indicates that the highest polymerization rate occurs during the first seconds and polymerization shrinkage increases steadily afterward, which is in agreement with the other study (Abbasi et al., 2018).

Although many resin composites are categorized as bulk‐fill composites, these materials show different behaviors, so it is vital to detect these materials to realize their features and behaviors. Considering that GBI is an approximately new composite and information about its composition and filler content has not been released, the energy dispersive X‐ray analysis is recommended.

Since our study was designed in‐vitro and clinical conditions cannot be precisely simulated in vitro, care must be taken to the generalization of the results to the clinical setting. Moreover, future studies are required to assess the wear resistance and fracture toughness of bulk‐fill composites, particularly GBI, in comparison with conventional composites.

## 5 CONCLUSION

Based on the results of this study, the recently introduced composite (GBI) had similar polymerization shrinkage compared to the conventional flowable one. Polymerization shrinkage of bulk‐fill composites was similar to or lower than conventional composites.

## AUTHOR CONTRIBUTIONS

*Conceptualization*: Somayeh Khoramian Tusi, Maryam Mohammadi Savadroodbari. *Methodology*: Maryam Mohammadi Savadroodbari, Hajar Hamdollahpoor. *Formal analysis*: Mahmood Sheikh Fathollahi. *Investigation*: Hajar Hamdollahpoor. *Writing–original draft preparation*: Hajar Hamdollahpoor. *Writing–review and editing*: Somayeh Khoramian Tusi, Mahmood Sheikh Fathollahi. *Supervision*: Somayeh Khoramian Tusi, Maryam Mohammadi Savadroodbari.

## CONFLICT OF INTEREST

The authors declare no conflict of interest.

## Data Availability

All data used to support the findings of this study are included in the ScholarOne system.
